# “Man, what took you so long?” Social and individual factors affecting adult attendance at voluntary medical male circumcision services in Tanzania

**DOI:** 10.9745/GHSP-D-12-00037

**Published:** 2013-03-21

**Authors:** Marya Plotkin, Delivette Castor, Hawa Mziray, Jan Küver, Ezekiel Mpuya, Paul James Luvanda, Augustino Hellar, Kelly Curran, Mainza Lukobo-Durell, Tigistu Adamu Ashengo, Hally Mahler

**Affiliations:** a Jhpiego Tanzania, Dar es Salaam, Tanzania; b U.S. Agency for International Development, Washington, DC, USA; c Department of Cultural Anthropology and Tourism, Tumaini University, Iringa, Tanzania; d Regional Medical Office, Ministry of Health and Social Welfare, Iringa, Tanzania; e Jhpiego, Baltimore, Maryland, USA; f Johns Hopkins Bloomberg School of Public Health, Baltimore, Maryland, USA

## Abstract

**Background:**

In 2009, the Government of Tanzania embarked on scaling up voluntary medical male circumcision (VMMC) services for HIV prevention in 8 priority regions, with the aim of serving 2.8 million boys and men ages 10–34 years by 2013. By mid-2012, more than 110,000 boys and men in Iringa and Njombe regions had received VMMC. The majority (85%) of these VMMC clients were under 19 years old (average age, 16 years). This study aimed to identify potential barriers and facilitators to VMMC among older men.

**Methods:**

We conducted 16 focus group discussions, stratified by sex and age, with 142 purposefully selected participants in 3 districts of Iringa and Njombe regions.

**Results:**

Both men and women generally had positive attitudes toward VMMC. Social and personal barriers to obtaining VMMC among adult men included shame associated with seeking services co-located with younger boys and perceived inappropriateness of VMMC after puberty, particularly after marriage and after having children. Additional barriers included concerns about partner infidelity during the post-surgical abstinence period, loss of income, and fear of pain associated with post-surgical erections. Facilitators included awareness of the HIV-prevention benefit and perceptions of cleanliness and enhanced attractiveness to women.

**Conclusions:**

While men and women in Iringa and Njombe regions in Tanzania generally view VMMC as a desirable procedure, program implementers need to address barriers to VMMC services among adult men. Selected service delivery sites in the Iringa and Njombe regions will be segregated by age to provide services that are “friendly” to adult men. Services will be complemented with behavior change communication initiatives to address concerns of older men, encourage women’s support for circumcision and adherence to the post-surgical abstinence period, and change social norms that inhibit older men from seeking circumcision.

## Abstract (precis)

In a study in Tanzania, men and women generally supported male circumcision; however, cultural values that the procedure is most appropriate before adolescence, shame associated with being circumcised at an older age, and concerns about the post-surgical abstinence period have led to low uptake among older men.

## INTRODUCTION

Voluntary medical male circumcision (VMMC) has been shown to be a safe and effective method of reducing female-to-male HIV transmission by about 60% in randomized controlled trials and by up to 73% in post-trial observation.^1-4^ Cost and impact modeling have shown that rapid scale up of VMMC among men ages 15–49 years would drastically reduce HIV transmission.^5^

We recognize 3 major contexts in Tanzania where male circumcision (MC) is performed:

1. Traditional MC—generally conducted around or before puberty as part of a traditional rite of passage
2. Religious MC—typically performed during infancy (primarily for Tanzanian Muslims)
3. Voluntary medical MC for HIV prevention—offered since 2009 as a free service through selected government health facilities

In Tanzania, where adult HIV and MC prevalence is 5.7% and 67%, respectively, the national HIV prevention strategy of the Ministry of Health and Social Welfare prioritized VMMC for boys and men ages 10–34 years, particularly in regions with high HIV prevalence and low MC prevalence.^6^ The proportion of boys and men ages 15–49 years who reported being circumcised ranged from 21% in Shinyanga to 100% in Lindi.^7^

Iringa Region, with the highest HIV prevalence in the country (15.7%) and one of the lowest circumcision levels (29%), has set a goal to circumcise 264,990 boys and men ages 10–49 years by 2015.^6^^,^^7^ Since this study's completion in 2011, the Iringa Region has been divided into two administrative regions, Iringa and Njombe. The populations of Iringa and Njombe have been estimated at 1,006,732 and 783,047, respectively.^8^ MC and HIV prevalence data in the newly defined regions are not currently available.

By July 2012, more than 110,600 male circumcision procedures had been conducted in Iringa and Njombe regions, with support from the Maternal and Child Health Integrated Program (MCHIP) funded by the U.S. Agency for International Development. This represents 42% of the initial regional goal for Iringa. The majority (n = 86,972) of VMMCs were conducted through a combination of high-volume outreach campaigns and static services during the cold season when demand is naturally high (cooler weather is thought to promote wound healing). It is estimated that 26% and 37% of uncircumcised men of all ages in Iringa and Njombe regions, respectively, have been reached through the VMMC program.^9^

In addition to the national age targets, the program has prioritized VMMC for older clients who are more likely to be sexually active and consequently at increased HIV risk. Reaching this subgroup of adult men (ages 20 and above) with VMMC would translate into the greatest HIV-prevention benefit in the near future. In the 2010 Tanzania Demographic and Health Survey, 76% of males ages 15–17 reported having never had sex; by ages 23–24 years, that prevalence declined to 14.3%.^10^

The VMMC program in Iringa and Njombe has extensively used radio coverage, billboards, and experiential media for demand creation and communication efforts. Messages communicate the HIV-prevention benefit of VMMC for all men but primarily focus on adult men. Communication materials and messages explaining benefits to women have also been circulated and aired. Despite the communication focus on older, sexually active men, 85% of VMMC clients in these regions were under 20 years of age (mean age = 16 years, standard deviation [SD] = 5.1, range = 1–76). Merely 6% of VMMC clients were 25 years or older.

Communication campaigns in Iringa and Njombe have focused on the benefits of VMMC for older men, but most VMMC clients are under 20 years of age.

The Kenyan VMMC program has reported a similar pattern of young VMMC clients.^11^ Research conducted in Kenya and Uganda in circumcision clinical trials and other settings corroborate this cultural preference for circumcision at a younger age.^12^^,^^13^ A study on acceptability of VMMC among a community in the Mara Region of northern Tanzania that traditionally circumcises showed a strong preference toward pre-pubescent circumcision.^14^ Although approximately 67% of Tanzanian boys and men ages 15–49 are circumcised, the major ethnic groups in Iringa—the Hehe- and Bena-speaking people—do not traditionally circumcise males.^7^ Ethnic groups that circumcise males in Tanzania typically do so as part of religious or ethnic rites of passage during infancy, adolescence, or pre-adolescence.^15^

Reports from other countries highlighted fear of pain during the procedure, painful erections after surgery, and delays in healing as deterrents to seeking VMMC.^16-18^ Other barriers to VMMC included lack of knowledge about the HIV-prevention benefit, concerns about income loss during the post-surgical recovery period, and the belief that MC is a cultural practice meant for other ethnic groups.^13^

It was unclear whether the deterrents documented in these other contexts also explained lower VMMC uptake in Iringa and Njombe. This study was conducted to explore VMMC perspectives and identify barriers and motivators by age and sex, particularly with implications for older (ages 20 and above) sexually active, married men in Iringa and Njombe.

## METHODS

### Recruitment/Sample

Between February 21–26, 2011, a total of 142 participants (n = 68 women, n = 34 men ages 18–29, and n = 30 men ages 30 and above) were enrolled in focus group discussions (FGDs, N = 16) stratified by age and sex. The 16 FGDs were conducted in 3 districts in the 2 regions: Iringa Municipal Council (n = 6), Mufindi District (n = 5), and Njombe District (n = 5). These districts were selected for the relatively high volume of VMMCs provided, and to understand urban, peri-urban, and rural differences. Participants and sampling locations were purposefully selected in close collaboration with local government Council HIV/AIDS Coordinators (CHACs). The CHACs posted announcements and communicated verbally to potential participants about the study and assembled participants prior to the FGD sessions. CHACs were instructed to select people who fit the sex and age strata and who could easily reach the venue for the FGD.

### Design

All FGDs were conducted in Kiswahili in spaces with audio privacy, with an average of 8 participants (range = 7–11), a facilitator, and an observer. Sessions were 1.5–2 hours long. Facilitators and observers were gender-matched to the focus group participants, but observers could be female for male FGDs. Sessions were recorded digitally, and extensive notes were taken (in both English and Swahili) to document complementary information. See the Table for a description of the composition of the FGDs. Participant inclusion criteria were:

**Table:** Table. Composition of Focus Group Discussions

Age and Sex	No. of FGDs	Average No. of Participants/FGD	Total No. of Participants
Younger men (18–29)	4	8	32
Older men (30 and above)	3	9	28
Women (18–39)	6	9	52
Mixed gender	3	10	30
**Total**	**16**	**9**	**142**

Abbreviation: FGD, focus group discussion.

1. Ages 18–39 for women, 18–29 for the younger male FGD, and 30 or above for the older male FGD
2. Living within walking distance (approximately 5 km) of the selected location
3. Available to participate during the allocated FGD time

We set different age strata for the male FGDs in order to solicit views that potentially differed by age, on the premise that men would be more comfortable discussing their views with peers. In addition to the FGDs, we also conducted one mixed-gender participatory group exercise in each study district (N = 30, 10 participants per district). We asked participants to complete life-cycle exercises whereby they described VMMC appropriateness over the course of a man's life and other cultural factors that could influence a man's decision to get circumcised. Participants diagrammed the lifespan of a male starting from infancy and discussed the appropriateness of VMMC at different stages in his life, including VMMC benefits and drawbacks, barriers, and facilitators for men seeking VMMC services.

FGD participants completed an anonymous questionnaire in Kiswahili on demographic information (age, sex, marital/relationship status, parental status, circumcision status, and age of circumcision for males). Female participants were not asked about their partners' circumcision status. Questions were read aloud to the group, and assistance was provided to participants who requested it (about 30% of participants needed assistance).

FGDs were not stratified by circumcision status since MC status was collected anonymously. A man could have self-disclosed his circumcision status during FGDs, but no probes were made to differentiate views of circumcised versus uncircumcised men.

Verbal consent was obtained at the beginning of each FGD, and participants consented not to relay group discussions externally. Regardless of whether they completed the exercise, participants received approximately US$3 for their time. No participant declined participation or rescinded informed consent throughout the study. Human subjects approval was obtained through the Johns Hopkins Bloomberg School of Public Health Institutional Review Board, with formal written support from the office of the Iringa Regional Medical Officer.

### Analysis

After checking for missing fields, quantitative data from the survey were entered into PASW® Version 16 statistical software and analyzed. Immediately following each FGD, the moderator/facilitator teams discussed and revised their notes and highlighted key points to explore further in the digital recordings. FGDs were transcribed in Kiswahili. All coding and thematic analyses were conducted manually in Kiswahili by 3 analysts, and key themes and passages were translated into English.

## RESULTS

About half of the participants in the 16 FGDs were men (74 of 142 total participants, or 52%). Men were slightly older than women (mean age of men = 32.6 years, range = 18–68 versus mean age of women = 29.2 years, range = 18–47). The majority of men and women were married (66% and 57%, respectively) and were parents (77% and 82%, respectively). Most men (72%) were circumcised by age 13.5 years (range = 2–25 years).

The following themes emerged during analysis:

- Knowledge and attitudes toward MC
- Norms around MC/VMMC, particularly in relation to age and sexuality (specifically, sexual function and ability to fulfill sexual obligations during abstinence/healing period, and, for older men, shame)
- Other barriers to VMMC, including penile injury
- Ideal VMMC service delivery modalities to attract older men

### Knowledge and Attitudes Toward VMMC

Almost all of the participants correctly described VMMC as removal of the foreskin. Many participants also spontaneously mentioned prevention of HIV transmission and good hygiene as benefits of VMMC. Participants' attitudes were generally positive toward VMMC, and male participants mentioned peer pressure, women's preferences, disease prevention, and cleanliness as key motivating factors in seeking VMMC. Women mentioned greater sexual pleasure as a VMMC benefit. Sexual and aesthetic appeal of VMMC resonated more among younger men and women in the FGDs.

### MC/VMMC Norms

In Iringa, perceptions of MC seem to be shifting, from the view that the procedure is foreign and undesirable to one that promotes cleanliness, prevents infection, and increases attractiveness to women. This shift in norms was most striking among younger male participants, one of whom stated:

When I started high school … many of the other students were circumcised, and I wanted to get circumcised as well. … My parents didn't understand me at all. They said, “In our society, we don't do that.” They forbade me.

The growing desirability for male circumcision in Tanzania reflects a shift in social norms, from being a foreign procedure to one that promotes cleanliness, prevents infections, and attracts women.

Eventually, this young man was circumcised. Another young male participant stated:

We were born in the 80s … it is unusual to find [uncircumcised] men … I really haven't come across them.

An older male participant identified greater social status with circumcision:

A circumcised man has a higher status … For us boys of higher [older] age [jokingly], you might hit on a woman, and if you are not circumcised and she finds out, she won't agree to go with you. And I think that shows the status of circumcision.

There was a prevailing notion of “modern” Tanzanian culture in which the majority of men are circumcised. Two younger male participants emphasized:

There is no traditional culture for MC, but in town it is accepted as normal practice now.

Many of us have been to town, and if people see that you are uncircumcised in town, they laugh at you.

### Age and Circumcision

Male and female participants almost unanimously articulated that it was best to perform VMMC before puberty. Most participants believed that VMMC clients in their 20s or 30s would be ridiculed or thought to be unusual and shameful. A participant in the Mufindi young men's group stated:

If you see an old man getting circumcised, you will have to laugh at him a little bit like, “Man, what took you so long?”

Another participant explained:

*There is fear and shame if you reach over 19 and you go to get circumcised. You will have to wear a kanga* [cloth wrap worn by Tanzanian boys following traditional circumcision] *during the healing period and everyone will know.*

Participants identified men ages 25 and older as being “too old” to be circumcised. However, the notion of an “older” VMMC client extended beyond age to include marital/relationship status and parental status. For example, an unmarried, childless man in his 20s seeking VMMC would be considered normal but not if he were married with children. Participants also described it as shameful for a man's children to hear that their father had been circumcised:

… My child hearing that I've gotten circumcised?! A shame!

Cultural values in Tanzania encourage male circumcision to be performed before puberty—well before marriage and parenthood.

The theme of social hierarchy, in which older adult men occupy a distinguished status that they reinforce with appropriate behavior, emerged. Perceived age-inappropriate behavior by older men (such as seeking VMMC) can be viewed as a threat to their status within the family and society and would bring shame—the most frequently mentioned barrier to VMMC for older men. See the box for detailed descriptions of shame related to older men seeking VMMC.

Male FGD participants noted that they would like to support their sons in getting circumcised and would be in a better position to do so if they themselves had been circumcised. Participants also recognized that men seeking circumcision can serve as a positive example to the household.

**Box. Themes of Shame Related to Older Men Seeking VMMC**

**Perspectives of Older Men**

- It is an inappropriate activity for the age, and it causes status loss and embarrassment; need to hide the procedure.
- An elder should respect himself, control his sexual desires, and prevent diseases even without male circumcision (MC).

**Perspectives of Younger Men**

- It is not appropriate for older men to undergo MC; a man who does it will be laughed at.
- Status restricts older men from sharing intimate issues in front of society, neighbors, younger people, and their children.
- It is an age-inappropriate activity (scornful description of how pitiful it would be to see an older man wearing the wrap—*kanga* or *kitenge*—that boys often wear after traditional circumcision).

**Perspectives of Women**

- Undergoing MC is not status-appropriate for older men.

**Perspectives of Both Men and Women**

- If a man has passed the appropriate age for MC, it is considered shameful to admit his “incomplete” status.

### Sexuality and Circumcision

Participants consistently described MC as more sexually desirable to women because of increased virility and a more attractive penis. A female participant stated:

A man who has been circumcised is better because he is cleaner, and making love is more pleasurable.

A man's circumcision status was largely viewed as public information. This response by a younger female participant illustrated how circumcision status might become public information:

I heard an argument between two women over a man. One of them said: “… I don't need him, now I've got one who is circumcised—he is better for me; he really satisfies me.”

Given the cultural preponderance of multiple concurrent partnerships (MCP), participants viewed VMMC within marriage as a favorable option for reducing risk of HIV acquisition. A female participant stated:

Our husbands are not trustworthy; it is better they are circumcised. At least we will get some protection.

Men shared concerns about women in MCP as well. Participants brought up the custom of *mafiga matatu*, a Kiswahili expression used throughout Tanzania translated as “three cooking stones.” The expression implies that just as one needs three stones to hold a pot over the fire, a woman needs multiple partners to be fulfilled. Female participants also noted that when a woman's partner is circumcised, she can simply shift to another *mafiga* while she waits for him to heal.

According to younger men, not fulfilling sexual obligations to a girlfriend could lead her to leave him since premarital relationships are considered transient and largely about sex. Some women shared this concern about younger women. Concerns about a wife seeking another partner during the abstinence period were less of an issue among older/married men but were widely noted among participants:

For a married man, you have to agree with your wife about the 6 weeks following circumcision. It is necessary that the wife is aware of the rules and agrees for her husband to get circumcised. Many women will not be able to wait and this makes married men afraid to seek circumcision.

Women also expressed concerns about abstinence but thought that their men would not be able to abstain. One young woman laughingly stated:

*That type of man is out there! He knows that the food is steaming hot, and on top of that there are hot peppers in it, but he keeps eating it even when the tears are running down his face.* (Group laughs and agrees.)

Both men and women expressed doubts about being able to adhere to abstinence guidelines in the post-surgical healing period.

### Sexual Function and Penile Injury

Fears of penile injury from erections in the immediate post-operative period also emerged as a potential barrier. In particular, participants described a fear of erections causing stitches to rupture, resulting in pain and delayed wound healing. A mixed-gender group stated:

At 14 years, he [VMMC client] will have a better understanding about cleanliness … and if he dreams [of] his girlfriend, he could get an erection which could cause the stitches to get stretched out and cause damage to the penis.

### Loss of Income

Concern about loss of income from absence on critical, income-generating days was frequently raised in FGDs and viewed as a major deterrent to seeking VMMC. A young male participant stated:

Even for those who see the importance of circumcision, the process of circumcision takes time. During that time you better stay home to take care of yourself and not work.

### VMMC Service Delivery for Adult Men

Four VMMC service delivery approaches were discussed in the FGDs:

- Extending hours to weekends and evenings
- Couples-oriented services
- Separate services for boys and men
- Services with only male health care providers

Participants unanimously preferred the model of separate services for adult men and boys. Participants repeatedly stated that mixing services for boys/adolescents and adult men was culturally inappropriate. In all FGDs, participants acknowledged that an older man would lose face if he met a younger man or boy in this setting. While some participants presented other modifications to service delivery models, no other model had uniform support. More education for female partners was raised as a good idea, but neither male nor female participants indicated a preference for couples-oriented services. Female participants indicated satisfaction in participating in the decision-making to seek circumcision but not to be present during the surgery. Some men raised concerns about the presence of female health care providers, especially younger male participants who tend to be shyer.

Focus group participants unanimously preferred having separate VMMC services for adult men and boys.

## DISCUSSION

VMMC is currently being scaled up in Iringa and Njombe, and thus knowledge about and acceptance of the procedure appears to be high in the communities. While people generally view the procedure as a desirable one, the positive attitudes have not yet translated into service uptake across all subgroups of men. Cultural and financial barriers, as well as perceptions about pain and injury, were major barriers for certain segments of the male population, particularly with regard to age and stage of life. Concerns about income loss during the post-surgical period were also pervasive. The most prominent barrier for adult men with children was shame associated with what they perceived as an age-inappropriate procedure.

The preference for pre-adolescent/pre-adult VMMC has been described in other studies.^12^^,^^14^^,^^19^ In Iringa and Njombe, FGD participants explained that adult men enjoy a privileged place in society; revealing the fact that they are uncircumcised would compromise their status.

The 6-week abstinence period was an important concern, particularly for men with younger female partners who were thought to have multiple partnerships. Many women felt that VMMC offered some protection against HIV if their male partners had multiple sex partners. Conversely, men were concerned that their female partners may be unfaithful during the post-circumcision abstinence period.

Findings from a study in Kenya showed that marital or cohabiting status strongly predicted early resumption of sex among VMMC clients.^12^ This study did not explore the reasons for early resumption of sex (for example, an individual's sexual desire versus a wish to fulfill sexual obligations to his partner), but based on our findings it might be important to investigate this issue further. Epidemiologic modeling studies have shown that the benefits of VMMC at the population level can be conferred to women, but the role of concurrency in mitigating the benefit as suggested by these participants has not been explored and warrants further study.^20^

Future research should explore how concerns about partner faithfulness during the post-surgical abstinence period or other factors can influence early resumption of sexual activity.

Relevant counseling and informational materials that address issues related to age of VMMC clients, seasonal timing of the procedure, and effects on sexuality—particularly factors influencing post-circumcision abstinence—will be developed.^21^

Demand creation, education, and service delivery models to promote VMMC uptake among adult males in Iringa will be adapted based on these study findings. As a result of this investigation, selected service delivery sites will segregate clients by age to decongest service delivery sites and hopefully attract older, married men and men who are parents—the type of clients who might be inhibited to undergo VMMC due to a lack of privacy. Static sites devoted to serving older men will be provided rather than using campaigns that tend to attract younger men and adolescents.

In Roger's diffusion of innovation theory—a framework explaining how new ideas are adopted—the pace of adoption of a new idea or process is informed by how people perceive a new behavior, whether people believe the new behavior has advantages over their current practice, how easy it is to practice the new behavior, whether the new behavior can be tried without risk, and people's perceptions of how others who have adopted the behavior have fared.^22^ The theory defines 5 categories of adopters of innovations, depending on how early they are willing to adopt a new idea. Applying this theory to the 110,000 VMMC clients in Iringa and Njombe (representing an estimated 30% of uncircumcised 10–39-year-old males in the regions), VMMC has passed the “early adopters” stage (the second category of innovators) and is well into the third “early majority” stage. Early adopters in Iringa and Njombe appear to be younger men and boys. To reach the last 2 adopter categories of “late majority” and “laggards,” messages and approaches used in the community and among the client base need to reflect concerns of older men, as well as strive to change ingrained social norms among secondary audiences, such as partners and community leaders.

Behavior change communication messages should reflect concerns about the appropriateness of the procedure later in life and the post-surgical abstinence period.

### Study Limitations

The self-reported prevalence of MC (72%) in the study sample was higher than the regional average (29%), suggesting that this study might not be representative of regional barriers and facilitators of VMMC. This difference in MC prevalence is not well understood, but we hypothesize that it could have resulted from selection bias, representative sampling from areas with higher MC prevalence than regional MC prevalence reported several years ago, or chance. This study sample was drawn from areas where the VMMC program is more mature and as a result might have higher MC levels than those measured in the 2007-08 Tanzania HIV/AIDS and Malaria Indicator Survey. Selection bias also could have resulted from the purposive selection and the inclusion criteria that participants must reside close to the study site.

Alternatively, social desirability could have played a role in the reported circumcision status. Even a small number of participants erroneously reporting being circumcised when they were not could dramatically skew the observed MC prevalence due to the small sample size. One participant did reveal during the FGD that he had marked that he was circumcised on the form when he actually was not. We tried to create an environment to enhance truthfulness by posing questions in third person to minimize bias. In addition, the questionnaire did not ask whether the male participants' circumcision procedures were performed medically or traditionally. Participants were not asked about their ethnicity; we assumed most were Hehe, the predominant ethnic group in Iringa and Njombe. However, in urban areas with high mobility, ethnic homogeneity cannot be assumed.

## CONCLUSION

To effect change in the community and interpersonal environment, community approaches should involve community leaders, champions, positive social deviants, peer educators, and social media. In addition, service delivery approaches should be appropriate to social norms. Women's support for circumcision, described by participants as key for both a man's decision to seek VMMC and his ability to adhere to post-circumcision abstinence guidelines, must be solicited through education and communication aimed at women. We recommend that VMMC scale up includes formative qualitative assessment of attitudes, care-seeking practices, and social norms related to VMMC, particularly the appropriate age and life stage to get VMMC, to help guide this process. The findings also highlighted the need to address concerns and misconceptions around erections during the post-circumcision healing period.

## References

[1] Auvert B. Taljaard D. Lagarde E. Sobngwi–Tambekou J. Sitta R. Puren A. Randomized, controlled intervention trial of male circumcision for reduction of HIV infection risk: the ANRS 1265 trial. PLoS Med. 2006;3(5), e298 16231970 10.1371/journal.pmed.0020298 PMC1262556

[2] Bailey R. C. Moses S. Parker C. B. Agot K. Maclean I. Krieger J. N. Male circumcision for HIV prevention in young men in Kisumu, Kenya: a randomised controlled trial. Lancet. 2007;369(9562), 643–656 10.1016/S0140-6736(07)60312-2 17321310

[3] Gray R. H. Kigozi G. Serwadda D. Makumbi F. Watya S. Nalugoda F. Male circumcision for HIV prevention in men in Rakai, Uganda: a randomised trial. Lancet. 2007;369(9562), 657–666 10.1016/S0140-6736(07)60313-4 17321311

[4] Gray R. Kigozi G. Kong X. Ssempiija V. Makumbi F. Wattya S. The effectiveness of male circumcision for HIV prevention and effects on risk behaviors in a posttrial follow-up study. AIDS. 2012;26(5), 609–615 10.1097/QAD.0b013e3283504a3f 22210632 PMC4296667

[5] Njeuhmeli E. Forsythe S. Reed J. Opuni M. Bollinger L. Heard N. Voluntary medical male circumcision: modeling the impact and cost of expanding male circumcision for HIV prevention in Eastern and Southern Africa. PLoS Med. 2011;8(11), e1001132 10.1371/journal.pmed.1001132 22140367 PMC3226464

[6] National AIDS Control Programme (NACP). National strategy for scaling up male circumcision for HIV prevention: enhancing men's role in HIV prevention [Internet]. Dar es Salaam, Tanzania: NACP 2010 Jun [cited 2012 Dec 1]; 48 p. Available from: http://www.nacp.go.tz/documents/Nationalstrategy.pdf

[7] Tanzania Commission for AIDS (TACAIDS), Zanzibar AIDS Commission (ZAC), National Bureau of Statistics (NBS), Office of the Chief Government Statistician (OCGS), and Macro International Inc. Tanzania HIV/AIDS and malaria indicator survey 2007–08 [Internet]. Dar es Salaam, Tanzania: TACAIDS, ZAC, NBS, OCGS, and Macro International Inc; 2008. [cited 2012 Dec 1] 297 p. Available from: http://www.measuredhs.com/pubs/pdf/AIS6/AIS6_05_14_09.pdf

[8] National Bureau of Statistics, Ministry of Planning, Economy and Empowerment. Iringa Regional and District Projections Volume XII [Internet]. Dar es Salaam, Tanzania: National Bureau of Statistics; 2006 Dec. [cited 2012 Dec 1] 250 p. Available from: http://www.nbs.go.tz/projections/iringa_projections.pdf

[9] Mahler H. R. Kileo B. Curran K. Plotkin M. Adamu T. Hellar A. Voluntary medical male circumcision: matching demand and supply with quality and efficiency in a high-volume campaign in Iringa Region, Tanzania. PLoS Med. 2011;8(11), e1001131 10.1371/journal.pmed.1001131 22140366 PMC3226544

[10] National Bureau of Statistics (NBS) [Tanzania] and ICF Macro. Tanzania Demographic and Health Survey 2010 [Internet]. Dar es Salaam, Tanzania: NBS and ICF Macro; 2011. [cited 2012 Dec 1] 482 p. Available from: http://www.measuredhs.com/pubs/pdf/FR243/FR243[24June2011].pdf

[11] Government of K. e. n. y. a., Ministry of Public Health and Sanitation, National AIDS/STI Control Programme. Progress report on Kenya's voluntary medical male circumcision programme, 2008-10 [Internet]. Nairobi, Kenya: Government of Kenya; 2012 Jan. [cited 2012 Dec 1] 32 p. Available from: http://www.malecircumcision.org/country_updates/documents/VMMC_2010_report_final.pdf

[12] Herman–Roloff A. Bailey R. C. Agot K. Factors associated with the early resumption of sexual activity following medical male circumcision in Nyanza Province, Kenya. AIDS Behav. 2012;16(5), 1173–1181 10.1007/s10461-011-0073-1 22052231 PMC3677080

[13] Westercamp N. Bailey R. C. Acceptability of male circumcision for prevention of HIV/AIDS in sub-Saharan Africa: a review. AIDS Behav. 2007;11(3), 341–355 10.1007/s10461-006-9169-4 17053855 PMC1847541

[14] Wambura M. Mwanga J. R. Mosha J. F. Mshana G. Mosha F. Changalucha J. Acceptability of medical male circumcision in the traditionally circumcising communities in Northern Tanzania. BMC Public Health. 2011;11:373 10.1186/1471-2458-11-373 21605433 PMC3112418

[15] Wambura M. Mwanga J. Mosha J. Mshana G. Mosha F. Changalucha J. Situation analysis for male circumcision in Tanzania: final report [Internet]. Mwanza and Dar es Salaam, Tanzania: National Institute for Medical Research and Ministry of Health and Social Welfare; 2009 Sep. [cited 2012 Dec 1] 152 p. Available from: http://www.malecircumcision.org/programs/documents/TanzaniaMaleCircumcisionSituationAnalysis_September_09.pdf

[16] Lukobo M. D. Bailey R. C. Acceptability of male circumcision for prevention of HIV infection in Zambia. AIDS Care. 2007;19(4), 471–477 10.1080/09540120601163250 17453585

[17] Ngalande R. C. Levy J. Kapondo C. P. N. Bailey R. C. Acceptability of male circumcision for prevention of HIV infection in Malawi. AIDS Behav. 2006;10(4), 377–385 10.1007/s10461-006-9076-8 16736112

[18] Bailey R. C. Muga R. Poulussen R. Abicht H. The acceptability of male circumcision to reduce HIV infections in Nyanza Province, Kenya. AIDS Care. 2002;14(1), 27–40 10.1080/09540120220097919 11798403

[19] Lundsby K. Draebel T. Wolf Meyrowitsch D. 'It brought joy in my home as in the area of my wife.' How recently circumcised adult men ascribe value to and make sense of male circumcision. Glob Public Health. 2012;7(4), 352–366 Epub 2011 Nov 17. 10.1080/17441692.2011.632638 22087766

[20] Layer E. H. Beckham S. W. Momburi R. B. Kennedy C. E. 'After my husband's circumcision, I know that I am safe from diseases': women's attitudes and risk perceptions towards male circumcision in Iringa Region, Tanzania. Paper presented at:19th International AIDS Conference; 2012 July 22–27; Washington, DC. Abstract available from:http://www.iasociety.org/Abstracts/A200744778.aspx

[21] Bronfenbrenner U. The ecology of human development: experiments by nature and design. Cambridge, MA: Harvard University Press; 1979

[22] Rogers E. Diffusion of innovations. 4th ed. New York: Free Press; 1995

